# Adolescents’ involvement in mental health treatment and service design: a systematic review

**DOI:** 10.1186/s12913-024-11892-2

**Published:** 2024-11-28

**Authors:** Petter Viksveen, Nicole Elizabeth Cardenas, Siv Hilde Berg, Anita Salamonsen, Julia Rose Game, Stig Bjønness

**Affiliations:** 1 Faculty of Health Sciences, University of Stavanger, Stavanger, Norway; 2 Department of Quality and Health Technology, Faculty of Health Sciences, University of Stavanger, Stavanger, Norway; 3 Faculty of Humanities, University of Copenhagen, Copenhagen, Denmark; 4 Faculty of Health Sciences, Regional Centre for Child and Youth Mental Health and Child Welfare - North, The Arctic University of Norway, Tromsø, Norway; 5 Department of Public Health, Faculty of Health Sciences, University of Stavanger, Stavanger, Norway

**Keywords:** Adolescents, Mental healthcare, Shared decision-making, Systematic review, User involvement, User participation

## Abstract

**Background:**

Adolescents’ involvement in their mental healthcare is considered a fundamental human right. However, there is a lack of consensus regarding the extent and nature of user involvement and limited research on user involvement in adolescent mental healthcare has previously been identified. Given the evolving focus on this area, this study explores the experiences with, the effectiveness of, and safety issues related to adolescents’ user involvement in mental healthcare.

**Method:**

We conducted a systematic review, updating our original review with current research evidence relating to adolescents’ involvement in mental healthcare at individual and organizational levels. Searches across six databases, screening of reference lists, and suggestions from experts within the field helped to identify 5,527 records, of which 251 full text articles were screened. Established guidelines were used for data extraction, critical appraisal, and reporting of results.

**Results:**

Collectively, the literature searches resulted in 36 eligible studies, of which 28 provided qualitative data and eight provided quantitative data. The quantitative studies identified the importance of personal help and online tools to support adolescents’ involvement in their mental healthcare. A few qualitative studies suggested shared decision-making is associated with improved self-reported mental health and treatment satisfaction. No studies focused on safety issues. A thematic synthesis of qualitative studies yielded four themes at the individual level and two themes at the organizational level. The findings highlight the growing recognition of adolescents' right to be involved and their capacity to take part in decision-making, emphasizing shared decision-making, two-way communication, and trust as key components of a collaborative relationship fundamental to user involvement. Further facilitators for user involvement at both individual and organizational levels are described.

**Conclusion:**

The significance of user involvement in adolescent mental healthcare is underscored by a sense of increased empowerment and services tailored to meet adolescents’ needs. The evidence gathered from qualitative studies suggests involving adolescents in their treatment contributed to greater motivation for treatment, higher attendance rates, and treatment continuation. User involvement should emphasize adolescents’ preferences and a collaborative relationship that incorporates shared decision-making. Further implications for future practice and research are discussed.

## Introduction

Adolescence is a coplex life stage that marks the transition from childhood to adulthood. It is often legally concidered to span from ages 13 to 18 and involves increasing autonomy [1]. Including adolescents in healthcare decisions adresses the gap in healthcare policies that historically cater to either children or adults, neglecting the unique needs of this population [2]. The involvement of children and adolescents in matters affecting their lives is considered a human right [3]. According to article 12 of the Convention on the Rights of the Child, all children who are capable of forming their own views should have the right to express themselves freely, and for their views to be given weight in accordance with their age and maturity. In 2002, the UN General Assembly further emphasized the rights of children and adolescents to be involved in decision-making processes [4].

Involvement of children and adolescents in mental healthcare may be termed user involvement. To date, no internationally agreed standards or guidelines explicitly define or describe adolescents’ involvement in their mental healthcare. Various recommendations have been put forward in some countries. For instance, the Canadian Paediatric Society recommended in 2004 that doctors should involve children, adolescents, and their families, by providing them with adequate and sufficient information and by encouraging their involvement in decision-making processes [5]. We apply the term user involvement as described by Tritter and McCallum [6]. It accommodates a dynamic process at various levels in which power to effect change is derived from collaboration and partnerships which may involve different categories of participants and different approaches of involvement.

Principles for user involvement have been integrated in different ways and to varying degrees in healthcare policies and legislation in some countries. As an example, Norwegian legislation introduced in 1999 highlights the right of children to be listened to and for their views and wishes to be given increasing importance in line with their age and development [7]. Despite the introduction of a pathway for mental healthcare in Norway in January 2019 which also aimed at strengthening user involvement, a service review revealed that service users received limited information about the services and doubted whether their input had been taken into account [8]. Although Australia adopted the UN Convention on the Rights of the Child in 1990, some have reported that children’s rights have been insufficiently implemented [9]. Efforts to address these shortcomings include the development of a charter on the rights of children and young people in healthcare services which also mentions the right to participate in decision-making in accordance with their capabilities [10].

Participating in decision-making could include shared decision-making, defined by Chambers ([11], p.1) as: *“[…] when two autonomous and uncoerced agents both commit to actions that neither has reason to want to change based on their understanding of anticipated outcomes given the situation at hand and of the intended actions of the other party.”* The term user involvement encompasses shared decision-making, but extends further, emphasizing various degrees of power distribution. User participation and user involvement are terms used interchangeably in the literature. For instance, giving adolescents the opportunity to influence the focus of conversations may be just as important as involving them in decision-making processes. Moreover, adolescents may also choose for their voice to be represented by others in meetings that are of importance to their mental healthcare, or they may participate on behalf of interest groups or organisations as part of the process of planning, delivering or reviewing mental health services. Accordingly, user involvement may take place at the individual level, affecting adolescents’ personal healthcare; at systems level, affecting the development, revision and assessment of mental health services regionally or nationally; and at the political level to influence policies, funding decisions and legislation [12–14].

In spite of decisions made nationally and internationally to prioritise adolescents’ involvement in their mental healthcare, our systematic review published in 2022 identified limited research evidence to describe the experiences and perspectives of adolescents, their families and health personnel on user involvement in adolescents’ mental healthcare [15]. This review presented a meta-synthesis describing user involvement at the individual level. However, the existing evidence was insufficient to draw firm conclusions regarding user involvement at the systems level or for the effectiveness and safety of user involvement.

Given that reviews can become outdated whithin two years, as noted by The Cochrane Collaboration [16] and the increasing international focus on user involvement, it is likely that new research has since emerged. For instance, the significant number of projects presented at the 6th International Conference on Youth Mental Health in 2022 [17] illustrates the growing attention to user involvement. These developments suggest that the field may have evolved, with additional research evidence adressing the gaps identified in our previous review. Moreover, there is a pressing need for further knowledge to inform healthcare policy to align with human rights. This systematic review therefore updates the current research evidence relating to adolescents’ involvement in mental healthcare at the individual and at systems level [1, 15]. The study aims to explore experiences with user involvement for adolescents’ in mental healthcare, as well as its effectiveness and any associated safety concerns.

## Methods

We conducted a systematic review of qualitative and quantiative studies reporting on user involvement for adolescent in mental healthcare. Predifined eligibility criteria, search strategies, guidelines for data extraction, critical appraisal, and reporting of results were equivalent to our systematic review published in 2022 [1, 15]. The PRISMA guidelines were used to report the results. The data synthesis was adjusted to integrate additional identified articles. We followed Robinson et al.’s [18] recommendations for integrating existing systematic reviews into new reviews. The six authors of this updated systematic review include four experienced researchers (PV, SHB, AS, SB) and two youth co-researchers (JRG, NEC). The two co-researchers have been involved in the research project since 2017, where ten adolescents have been involved in setting research priorities; planning research (including this systematic review); developing and recruiting participants for cross-sectional surveys; analysing results of the research; adademic dissemination and non-academic communication of results in journals, in the media and at national and international conferences; and by developing (successful) funding proposals [19].

### Inclusion criteria

Inclusion criteria in the update review were identical to the criteria applied in the systematic review published in 2022 [15]. The criteria are presented in Table 1 and include a broad definition of the term *“user involvement”* to reduce the chance of excluding any potentially relevant research evidence. As in our previous review, the perspectives of multiple stakeholders were included (adolescents, parents/legal guardians, and health personnel), as long as these stakeholders reported on involvement of adolescents in mental healthcare; and not, for example, involvement of parents in adolescents’ mental healthcare, as this was not the focus of this updated systematic review. Moreover, the review did not include involvement of adolescents in planning, implementation or evaluation of research. The term *“user involvement”* was interpreted as more than “simply” attending therapy sessions, but required more active involvement in the planning, implementation, or review of adolescents’ mental healthcare, through processes which could also include shared decision-making. Where the systematic published in 2022 review was limited to the period from 2002 to 2019, the update search focused on the empirical research literature published from 2019 to 2022.

**Table 1:** Article inclusion criteria

Inclusion category	Category description	Notes
Adolescents	Majority within age range 13–18 years (MeSH Unique ID: D000293)	Included if more than 50% of the participants were within the age range
Study participants	Any participants reporting on adolescents’ involvement in mental healthcare	E.g. adolescents, caretakers, healthcare professionals
Mental healthcare	Healthcare services providing preventive or therapeutic interventions for diagnosed or self-reported mental health and/or substance use problems	Based on MeSH Unique ID: D003191
User involvement (individual level)	Involvement of the individual adolescent in her or his own mental healthcare	Experiences, views and wishes to plan, deliver, review or make other decisions affecting adolescents’ mental healthcare
User involvement (organizational level)	Adolescents’ experiences, views and wishes used to plan, deliver or review mental health services for adolescents in general, including to develop new or to improve existing services	Including adolescents’ experiences with mental health services used in practice implementation or testing in research
Research methods	Studies using qualitative, quantitative or mixed methods	
Publication types	Peer-reviewed publications	Grey literature such as academic theses and dissertations; conference abstracts, and proceedings were excluded from the update search
Languages	English, German, French, Danish, Norwegian, Swedish	
Publication year	2019– 2022	

Although young persons are still under development both biologically and socially at the age of 18 years, the age limit for adolescence is set to 18 in most legal systems and mental healthcare services worldwide. At the age of 18, young persons become independently responsible for their actions and even though treatment of young persons may continue within the context of child and adolescent mental health services within some countries, they are in most countries moved to adult mental healthcare. Moreover, for the most part, at this timepoint they are also given the right to make decisions about their own health independent of their parents’ involvement.

### Search strategy

The search strategy mirrored that of the original review, including a broad range of search terms and involving two researchers in all phases of the literature search. However, we limited the number of databases based on our previous experience to the following: PsycINFO, EMBASE, MEDLINE, PubMed, British Nursing Index (BNI) and Scopus. These databases were selected because they yielded most of the articles (*n = *22) in the original literature search. Only three titles in the original literature search were found through other databases. We identified the remaining articles by contacting other researchers who had previously published research in the same field and by searching reference lists of included articles. For the current update review, we did not carry out a Google Scholar search to identify articles in the grey literature, nor did we contact mental health organizations, as these two approaches did not result in additional articles in the systematic review published in 2022.

In accordance with the suggestions put forth by Robinson et al. [18], we searched for new reviews related to the same topic during our update review. However, we were unable to locate any relevant reviews apart from the systematic review published in 2022 [15]. Nor did we identify any new articles reporting on user involvement at the organizational level.

We used a broad range of search terms (Table 2), identical to terms used in the systematic review published in 2022. Searches were customized to fit with individual databases, to maximise search sensitivity and specificity. Searches were carried out in March 2022 by the first author (PV) and checked by the last author (SB).

**Table 2:** Literature search strategy

**Databases**	British Nursing Index, EMBASE, MEDLINE, PsycINFO, PubMed, Scopus
**Other sources**	Researchers: authors of included articles were contacted Hand search of reference lists of reviews and included articles
**Search terms 1**: Subject & MeSH terms	**User group & field of health**: adolescent psychiatry; adolescent psychology **Field of research**: clinical decision-making; community participation; consumer participation; cooperative behavior; decision making; decision making, organizational; information dissemination; information sharing; patient participation; personal autonomy, public opinion; self-determination
**Search terms 2**: Title search terms	**User group**: adolescents; teenagers; youth **Field of health**: mental; psychology; psychiatry
	**Field of research**: autonomy; client-centred; collaboration; consultation; contribution; decision-making; empowerment; engagement; governance; inclusion; information sharing; involvement; mutual agreement; negotiation; opinions; participation; partnership; patient-centred; peer support; perspectives; self-determination

During the screening process, a total of 550 new titles and abstracts were evaluated. Out of these, 528 were excluded, whereas the full text of the remaining 22 articles was considered by the two lead authors (PV and SB). Five new articles were added to the update review after mutual agreement on inclusion and exclusion. Together with the original literature search, a total of 5 527 articles were screened, with 251 full text articles considered. Collectively, the two literature searches resulted in 36 articles that could be included for further analysis in the systematic review. Data records were managed using Endnote (version 20.4.1).

### Data extraction

The guidelines used for data extraction were identical to the original literature review, including the Critical Appraisal Skills Programme (CASP) for qualitative studies [20], and the STROBE statement checklist for cohort, case–control and cross-sectional studies [21]. These guidelines covered the articles identified through the update search. Additionally, the Cochrane Consumers and Communication Review Group’s data extraction template for trials was used in the original literature search [21]. Data extraction was carried out by one out of six researchers, and checked by a second researcher or a youth co-researcher. There were no discrepancies in the assessments. For studies using quantitative methods, main outcomes were reported. In the event of multiple outcomes, only those relevant to the systematic review were included.

### Quality appraisal

Quantitative studies were evaluated for risk of bias using the Cochrane Collaboration's guidelines, which assessed the risk of selection, performance, detection, attrition, and reporting bias [22]. Confounding factors were also considered. The Pragmatic Explanatory Continuum Indicator Summary (PRECIS) tool was used to evaluate the applicability and generalizability of the results [23]. Qualitative studies were appraised using the Critical Appraisal Skills Programme (CASP) to assess rigor, credibility, and relevance [20]. Each CASP item was scored as satisfactory ("yes"), not satisfactory ("no"), or providing insufficient information to be assessed ("unclear"). Study quality categories were determined based on the number of items considered to be satisfactory. Articles published by authors of the systematic review were assessed by other researchers.

### Reporting of results

**Fig. 1:**
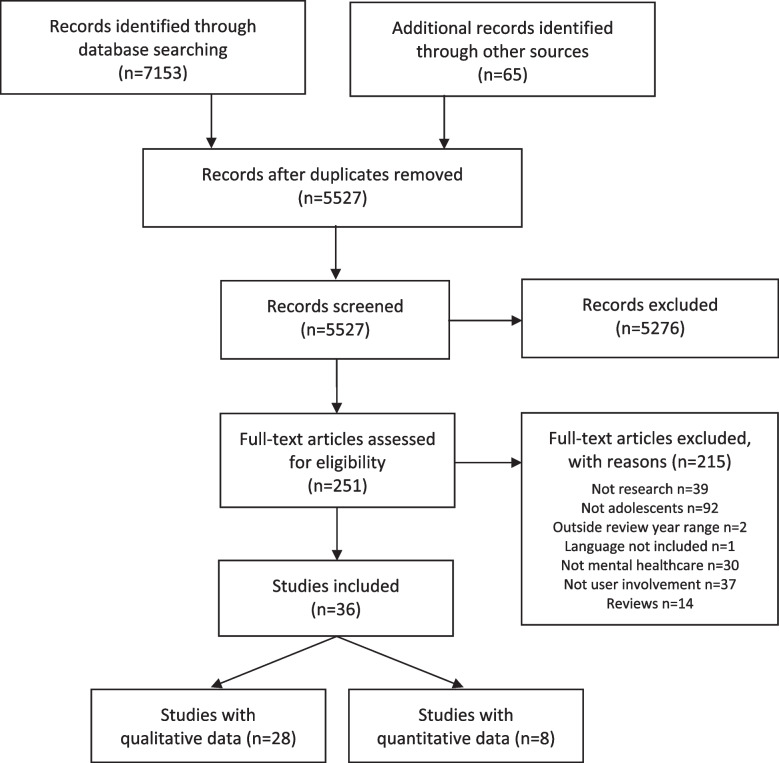
Systematic review PRISMA flow diagram

The Preferred Reporting Items for Systematic reviews and Meta-Analyses (PRISMA) flow diagram [24] was used to provide an overview of the studies evaluated collectively for the original and updated searches (Fig. 1). However, key numbers from the update search are presented separately in text. The STROBE statement was used to report observational (cross-sectional) studies [21], and the CASP checklist for qualitative studies [20]. No studies using other research designs were identified through the update search.

The characteristics of studies were tabulated and included participant details (age, gender, adolescents’ mental health status and whether the condition had been diagnosed); the category of persons reporting (adolescents, parents/guardians, or staff); the level of involvement (either individual or organizational); intervention/treatment and study setting; research methods, and overall quality assessment.

### Synthesis methods

Due to the heterogeneity of the studies regarding methodology and outcome measures, a statistical meta-analysis was considered inappropriate, and quantitative data are presented in a table with a narrative summary. For the single study using a quantitative design, an assessment of risk of bias [22] was carried out, as well as external validity, using the PRECIS guidelines to assess the pragmatic-explanatory continuum [23].

For studies using qualitative research designs we conducted a thematic synthesis according to Thomas & Harden [25] to report on experiences with involvement of adolescents in their mental healthcare (user involvement at the individual level). As for our the systematic review published in 2022, we analyzed results across different contexts and participants, to attempt to develop new explanations, constructs or hypotheses [25, 26]. The analysis draws on techniques used in thematic analysis, which goes beyond the original content of the original studies, and it suggests that the synthesis is more than merely the sum of the individual studies [27].

We used the thematic synthesis from the systematic review published in 2022 as a starting point for the update analysis. The first author (PV) and a youth co-researcher (NEC) carried out the initial updated thematic analysis. The inclusion of the youth co-researcher contributed to ensure that adolescent perspectives were included in the analytic process. The original synthesis was adjusted to integrate the content of the newly identified articles. As part of this analysis, we considered whether the research literature identified through the update search agreed with (convergence), complemented (complementarity), or contradicted (contradiction) the results of the thematic synthesis developed through the systematic review published in 2022 [15]. The new literature complemented three themes reporting on user involvement at the individual level (unilateral clinician control versus collaborative relationship, capacity and support for active involvement, the right to be involved). The titles of two themes were adjusted and their contents were considerably expanded, and the new information resulted in development of three sub-themes for two of the themes, as well as development of one new theme. Moreover, some of the new findings confirmed previous descriptions of themes (convergence).

## Results

This updated systematic review provides evidence-based knowledge from 36 studies reporting on user involvement in adolescent mental healthcare. Twenty-eight studies included qualitative data, whereas eight used quantitative research methods. The updated thematic synthesis offers a more extensive description of user involvement at the individual level, compared to the systematic review published in 2022 [15]. A single study using quantitative methods adds some insight into the effectiveness of user involvement at the individual level. No new studies reported on user involvement at the organizational level or the safety of user involvement.

### Characteristics of qualitative studies

The review included 28 qualitative studies with a total of 643 participants (Table 3). The median number of participants was 21 (interquartile range 14–30). Most participants were adolescents (79%), while parents/guardians and healthcare staff accounted for 13% and 7%, respectively. Studies were conducted in various primary and secondary healthcare settings. The gender distribution varied considerably among the studies, with 20% to 100% being female. However, the overall proportion of males and females was equal. Most studies (*n = *17) focused on adolescents with diagnosed mental health conditions or self-reported mental health problems such as depression, eating disorders, and Attention Deficit/Hyperactivity Disorder (ADHD). The remaining 11 studies did not specify mental health problems. In terms of user involvement, 21 studies reported involvement at the individual level, 11 studies reported involvement at the organizational level, while four of these studies reported involvement at both levels. Additional characteristics of the studies can be found in Table 3.

**Table 3:** Characteristics of qualitative studies

Reference	Participant characteristics ^a^	Intervention/treatment, study setting	Methods ^b^	Involvement level	Quality assessment (CASP)
Bjønness 2015 [28], Norway	*N = *14, age ≥ 16 years (girls x̄ = 18.2, boys x̄ = 17.3), female 64%, mental health conditions (unspecified)	Treatment unspecified, child and adolescent outpatient mental health services, specialist care	Qualitative study Convenience sample, recruited by health personnel Semi-structured interviews Systematic text condensation	Individual	High
Bjønness 2020 [29], Norway	*N = *15, health personnel/managers, female 53%, all with CAMHS experience, median 13 years (range 9 months to 35 years)	Child and Adolescent Mental Health Service (CAMHS) inpatient hospital settings for adolescents 13–18 years, specialist care	Qualitative study Purposive sample Focus group interviews (*n = *3) Systematic text condensation	Individual	High
Bjønness 2020 [30], Norway	*N = *10, age 16–18 years, female 80%, mental health conditions: anxiety (*n = *5), depression (*n = *5), autistic spectrum disorder (*n = *2), ADHD (*n = *3), psychosis (*n = *2), eating disorders (*n = *1), trauma/PTSD (*n = *1), not stated (*n = *1)	Child and Adolescent Mental Health Service (CAMHS) settings, specialist care	Qualitative study Purposive sample, recruited by chief physician/psychologist Semi-structured interviews Phenomenological hermeneutic 6-stage approach	Individual	High
Bjønness 2022 [31], Norway	*N = *12, parents of children age x̄ = 17.5 (median 17, range 13–22), mothers 75%, mental health conditions (unspecified)	Treatment unspecified, child and adolescent outpatient mental health services, specialist care	Qualitative study Purposive sample, recruited by CAMHS managers Semi-structured interviews (individual *n = *8, couples *n = *2) Thematic content analysis	Individual	High
Block 2013 [32], USA	*N = *25, age 12–17 years, female 44%, mental health conditions (unspecified)	Treatment unspecified, outpatient mental health services	Qualitative study Consecutive sample referred from schools to mental health services, participation rate: 32% (25 of 78) Semi-structured interviews Grounded theory analysis	Individual	Moderate
Boydell 2010 [33], Canada	*N = *30, age 13–18 years (*n = *19), 7–12 years (*n = *11), female 43%, conditions: ODD, ADHD, mood disorder, learning disability, anxiety disorder, conduct disorder, attachment disorder, developmental disability, foetal alcohol effects, adjustment disorder	Psychiatric consultations using interactive video conferencing technology, University division of child psychiatry with training sites at children’s, teaching & community hospitals	Qualitative study Recruitment strategy not specified Individual interviews Interpretive interactionist framework analysis	Individual & Organisational	Moderate
Coates 2014 [34], Australia	*N = *12, age 15–23 years (x̄ = 18.9), female 58%, conditions: anxiety, depression, PTSD, eating disorder, borderline personality disorder	Treatment unspecified, offered by foundation providing services for youth with mental health and/or drug and alcohol issues, governed under services provided by the local health district, primary care	Qualitative study Recruitment of new youth alliance members joining a national youth mental health foundation through advertising and information sessions Focus group interview Analytic approach not described	Organisational	Moderate
Coates 2016 [35],^**c**^ Australia	*N = *15, adolescents *n = *12, managers *n = *3, adolescents: age 15–23 years (x̄ = 18.9), female 58%, conditions: anxiety, depression, PTSD, eating disorder, borderline personality disorder	Treatment unspecified, offered by foundation providing services for youth with mental health and/or drug and alcohol issues, governed under services provided by the local health district (primary & secondary care)	Qualitative study Recruitment of new youth alliance members joining a national youth mental health foundation through advertising and information sessions Focus group interviews: adolescents (*n = *3), management (*n = *1), supplemented with documents including model descriptions and youth activity logs Thematic analysis	Organisational	Moderate
Coyne 2015 [36], Ireland	*N = *47, adolescents *n = *15, parents *n = *32, adolescents: age 11–17 years, female 60%, conditions: mood disorder, ADHD, impulse control, anxiety, adjustment and behavioural disorders	Treatment unspecified, provided in three Child and Adolescent Mental Health Services (CAMHS) clinics	Qualitative study Recruitment by a clinician within the service Individual and focus group interviews Thematic analysis	Individual	High
Crickard 2010 [37], USA	*N = *17, adolescents *n = *6, parents/guardians *n = *6, staff *n = *5, adolescents: age 14–17 years, gender not specified, mental health conditions (unspecified)	Treatment unspecified, community mental health centre	Qualitative study Recruitment not described Individual interviews Analytic method not reported	Individual Organisational	Low
Forchuk 2016 [38], Canada	*N = *46, adolescents *n = *37, care providers *n = *9, adolescents: age 16–21 years (x̄ = 17, SD1.4), female 73%, conditions: symptoms of depression, comorbidities: anxiety disorder, mood disorder, eating disorder, psychotic disorder, personality disorder	Web-based application that allows adolescents to create and manage an electronic personal health record	Mixed methods, but only qualitative data used for the systematic review Recruitment through care providers working in acute and tertiary care facilities Focus group interviews Thematic analysis according to Leininger’s phases of qualitative data analysis	Organisational	Moderate
Graham 2014 [39], UK	*N = *50, age 16–25 years (16-17y *n = *22, 16-19y *n = *6), within in review age range: female 54%, unspecified self-reported mental health problems in 46% (*n = *13)	Treatment unspecified, various service use, primary care	Mixed methods, but only qualitative data used for the systematic review Snowballing recruitment through two GP practices, three CAMHS, student counselling service, homeless shelter, supported housing project Focus group and individual interviews, participatory research groups, nominal group technique Thematic analysis and nominal group technique	Individual	High
Gros 2017 [40], Canada	*N = *6, age 13–18 years, female 67%, conditions: psychosis, mood disorders, borderline personality disorders, eating disorders, suicide risk	Treatment unspecified (min. 3 days) in acute inpatient psychiatric unit and in a day unit	Qualitative study Convenience sampling Semi-structured interviews and observations of participants’ non-verbal behaviour and contextual information Constant comparative analysis method	Individual Organisational	High
Hart 2005 [41], UK	*N = *27, age 11–18 years, female 59%, conditions: depression, school behavioural difficulties, ADHD, self-harm, family breakdown	Treatment unspecified, child and adolescent mental health services (range: < 1 year to 8 years), primary care	Qualitative study Recruited by health personnel in specialist CAMHS Home interviews with adolescents & their parents, and focus group interviews (girls, boys & parents separately) Analysis method unclear, possibly thematic	Individual	Moderate
Hayes 2019 [42], UK	*N = *19, adolescents: *n = *9, mothers *n = *10, adolescents: age 12–17 years (x̄ = 14.5), female 67%, conditions: anxiety *n = *4, depression *n = *2, self-harm *n = *2, unspecified *n = *2	Outpatient clinical settings	Qualitative study Recruitment from health professionals and posters placed in waiting rooms Interviews: adolescents + parents (adolescents’ choice) Thematic analysis, Theoretical Domains Framework	Individual	High
Latif 2017 [43], UK	*N = *11, adolescents: *n = *4, nurses *n = *7, adolescents: age 10–18 years (x̄ = 15), female 100%, self-harm injuries	Treatment unspecified, inpatient acute care services/hospital	Qualitative study Recruitment from CAMHS Workshops with story boards Delphi technique	Organisational	Moderate
LeFrancois 2007 [44], UK	N not specified, age 11–18 years, gender not specified, mental health conditions unspecified	Treatment unspecified, adolescent psychiatric inpatient unit/hospital	Qualitative study Recruitment method unclear Semi-structured and unstructured individual and group interviews, adolescents’ self-recorded unstructured conversations, additional written material (e.g. personal diaries, poetry, cards, drawings), over 4 months Ethnographic study, analysis method unclear	Individual	Low
LeFrancois 2008 [45], UK	N not specified, age 11–18 years, gender not specified, mental health conditions unspecified	Treatment unspecified, adolescent psychiatric inpatient unit/hospital	Qualitative study Recruitment method unclear Semi-structured and unstructured individual and group interviews, observation of conversations and interactions between health personnel and adolescents, investigation of written material (e.g. patient files, diaries, internal policy documents), over 4 months Ethnographic study, analysis method unclear	Individual	Moderate
Manning 2016 [46], UK	*N = *8, age 10–18 years, gender not specified, conditions: self-harm, eating disorders	Treatment unspecified, acute inpatient care for adolescents with mental health problems, psychiatric unit/hospital	Qualitative study Recruitment from a tertiary children’s hospital, recruitment rate 13% (8 out of 63 invited) Nominal group technique: Participant generated statements related to their experiences Thematic analysis	Individual	Moderate
Moses 2011 [47], USA	*N = *80, age 13–18 years (x̄ = 15.6), female 61%, hospitalization reasons: suicidal ideation or non-suicidal self-harm (63%), suicide attempts (19%), aggression or out-of-control behaviour incl. substance use (13%), medication assessment or school refusal (6%)	Treatment unspecified, psychiatric inpatient treatment/hospital	Qualitative study Recruitment through hospital admission staff Semi-structured individual interviews Thematic analysis with constant comparative method	Individual	High
Nadeau 2017 [48], Canada	*N = *15, adolescents *n = *5, parents *n = *5, clinicians *n = *5, adolescents: age 12–17 years (x̄ = 13.6, SD2.0), female 20%, conditions: emotional external behaviour problems, depression, ADHD	Treatment unspecified, free local community health centres (CLSC)	Qualitative study Recruitment through primary care clinicians Semi-structured individual interviews Thematic analysis	Organisational	Moderate
Oruche 2014 [49], USA	*N = *24, adolescents *n = *12, caregivers *n = *12, adolescents: age 13–17 years, gender not specified, mental health treatment, conditions unspecified	Treatment unspecified, community mental health centre, primary care	Qualitative study Recruitment through community mental health centre, recruitment rate: 60% (12 of 20) Focus group interviews with adolescents (*n = *2) and caregivers (*n = *2) (separately) Content analysis	Individual	Moderate
Ranney 2015 [50], USA	*N = *21, age x̄ = 15.3 years, female 57%, depression symptoms (PHQ-9 x̄ = 11.3, SD6.5) and peer violence (CTS-2 x̄ = 11.0, SD9.5)	Text-message-based depression prevention intervention, primary & secondary care	Qualitative study Recruitment of consecutive adolescents at trauma pediatric emergency department, children’s hospital Semi-structured individual interviews Thematic analysis	Individual	High
Rodarmel 2014 [51], USA	*N = *30, age 14–21 years, gender not specified, mental health conditions unspecified	Treatment unspecified, school-based mental health services, primary care	Qualitative study Recruitment through school-based hospitalisation services (*n = *26), and youth involvement and family-school-community partnership groups (*n = *4) Open-ended narrative surveys Grounded theory study	Individual Organisational	Moderate
Stockburger 2005 [52], Canada	*N = *21, age 15–19 years, gender not specified, experiences with drugs and alcohol	Treatment unspecified, local drug and alcohol treatment and support programs	Qualitative study Recruitment through local youth-serving agencies Focus group interviews (*n = *4) Thematic analysis	Organisational	Moderate
Sundar 2012 [53], Canada	*N = *25, adolescents *n = *13, health personnel *n = *12, adolescents: age 16–20 years, female 62%, mental health conditions unspecified, use or have used mental health services	Treatment unspecified, mental health services, primary & secondary care	Qualitative study Recruitment of convenience sample, recruitment method not reported Focus group interviews with youth (*n = *2) and health personnel (*n = *2) Grounded theory approach, constant comparison method	Individual	High
Thorsen 2018 [54], USA	*N = *41, age 13–17 years; group A: *n = *20, age x̄ = 15.4 (SD1.4), female 100%, group B: *n = *21, age x̄ = 15.3 (SD1.2), female 43%; at risk of depression and victim or perpetrator of physical peer violence	Preventive CBT-based SMS-delivered intervention, emergency department in children’s hospital	Qualitative study Recruitment from an urban emergency department Semi-structured interview Thematic analysis	Organisational	Moderate
Wisdom 2006 [55], USA	*N = *22, individual interviews *n = *15: age 14–19 years (x̄ = 16.3), female 53%, focus group participants *n = *7: age 15 years, female 71%, diagnosis: major depression, dysthymia or depression not otherwise specified	Current or past psychotherapy and/or antidepressants, or no treatment, primary care	Qualitative study Recruitment: individual interviews through primary care personnel, focus group interview: through a high school Individual (*n = *15) and focus group interviews (*n = *1) Grounded theory approach, constant comparison method	Individual	High

^a^Participant characteristics includes age, gender, mental health status/conditions/problems

^b^Methods include research design, recruitment methods (for adolescents), data collection and analytic method. The reported design refers to the approach used to collect data of relevance to the review

^c^The first focus group interview included in Coates 2016 [35] was also reported on in Coates 2014 [34]

### Quality assessment of qualitative studies

All studies met the initial two criteria outlined in the Critical Appraisal Skills Programme (CASP) guidelines [20] by havincg a clear research aim and the appropriateness of using qualitative methodology to address the research objective (Table 4). The CASP guidelines recommend to proceed with an evaluation of the remaining questions after fulfilling these initial criteria. Overall, the majority of studies were of moderate (*n = *14) or high (*n = *12) quality, whereas two studies were of low quality [37, 44]. The most common limitation across the studies was the lack of consideration or reporting of the relationship between the researchers and the participants. Adequate description of this aspect was only found in six studies. Other prevalent weaknesses included insufficient rigor in reporting of data analysis methods (*n = *11), participant recruitment strategies (*n = *9), and consideration of ethical issues (*n = *7).

**Table 4:** Quality assessment of qualitative studies

Main author, year	1	2	3	4	5	6	7	8	9	10	Involvement level ^a^	Assessment (CASP) ^b^
Bjønness 2015 [28]	Y	Y	Y	Y	Y	Y	Y	Y	Y	Y	I	High
Bjønness 2020 [29]	Y	Y	Y	Y	Y	N	Y	Y	Y	Y	I	High
Bjønness 2020 [30]	Y	Y	Y	Y	Y	Y	Y	Y	Y	Y	I	High
Bjønness 2022 [31]	Y	Y	Y	U	Y	Y	Y	Y	Y	Y	I	High
Block 2013 [32]	Y	Y	Y	Y	Y	U	Y	U	Y	U	I	Moderate
Boydell 2010 [33]	Y	Y	Y	U	Y	U	N	Y	Y	Y	I/O	Moderate
Coates 2014 [34]	Y	Y	U	Y	Y	U	Y	U	Y	U	O	Moderate
Coates 2016 [35]	Y	Y	Y	Y	Y	N	Y	U	Y	Y	O	Moderate
Coyne 2015 [36]	Y	Y	Y	Y	Y	N	Y	Y	Y	Y	I	High
Crickard 2010 [37]	Y	Y	Y	U	Y	U	N	U	N	Y	I/O	Low
Forchuk 2016 [38]	Y	Y	Y	U	Y	U	U	U	Y	Y	O	Moderate
Graham 2014 [39]	Y	Y	Y	Y	Y	Y	Y	Y	Y	Y	I	High
Gros 2017 [40]	Y	Y	Y	Y	Y	Y	Y	Y	Y	Y	I/O	High
Hart 2005 [41]	Y	Y	Y	Y	Y	N	Y	U	Y	Y	I	Moderate
Hayes 2020 [42]	Y	Y	Y	U	Y	N	Y	Y	Y	Y	I	High
Latif 2017 [43]	Y	Y	Y	U	Y	U	Y	U	Y	Y	O	Moderate
LeFrancois 2007 [44]	Y	Y	Y	U	Y	U	N	U	Y	U	I	Low
LeFrancois 2008 [45]	Y	Y	Y	U	Y	U	N	U	Y	Y	I	Moderate
Manning 2016 [46]	Y	Y	Y	Y	Y	N	N	U	Y	U	I	Moderate
Moses 2011 [47]	Y	Y	Y	Y	Y	N	Y	Y	Y	Y	I	High
Nadeau 2017 [48]	Y	Y	Y	Y	Y	U	Y	Y	Y	U	O	Moderate
Oruche 2014 [49]	Y	Y	Y	Y	Y	U	Y	Y	Y	U	I	Moderate
Ranney 2015 [50]	Y	Y	Y	Y	Y	N	Y	Y	Y	Y	I	High
Rodarmel 2014 [51]	Y	Y	Y	Y	Y	NA	Y	Y	Y	U	I/O	Moderate
Stockburger 2005 [52]	Y	Y	Y	U	Y	Y	Y	U	Y	Y	O	Moderate
Sundar 2012 [53]	Y	Y	Y	Y	Y	N	Y	Y	Y	Y	I	High
Thorsen 2018 [54]	Y	Y	Y	Y	Y	U	N	Y	Y	Y	O	Moderate
Wisdom 2006 [55]	Y	Y	Y	Y	Y	U	Y	Y	Y	Y	I	High

^a^I = Individual level, 0 = Organizational level. ^b^CASP criteria are presented in appendix X. Y = Yes, *N = *No, U = Unclear, NA = Not applicable. Scoring: Low: Studies meeting 0–5 of the CASP checklist criteria. Moderate: studies meeting 6–8 of the criteria. High: studies meeting 9–10 of the criteria. For question 10, the score was considered to be Yes if the study was considered to be of "relevance" or "some relevance" to the systematic review, and Unclear if it was considered to be of "limited relevance"

### Experiences with user involvement – results of thematic syntheses

Thematic syntheses of qualitative studies were carried out separately for user involvement at the individual (Table 5) and at the organizational level. The thematic synthesis of qualitative studies at the individual level resulted in four themes: 1) The right to be involved; 2) Collaborative relationship; 3) Capacity and support; and 4) Outcomes of user involvement. The second and third theme, which are the most comprehensive, are each organized in three sub-themes (Table 5). At the organizational level the results consist of two themes: 1) Involvement outcomes relevant to adolescents’ needs; and 2) Conditions for optimal involvement. Each theme is described and references to the research literature are provided.

**Table 5:** Experiences with user involvement at the individual level

Themes	Sub-themes
The right to be involved	
Collaborative relationship	Information and two-way communication Trust and interpersonal chemistry Shared decision-making
Capacity and support	Adolescents’ capacity to be involved Health personnel’s capacity to facilitate involvement Support for adolescents’ involvement
Outcomes of user involvement	

#### User involvement at the individual level

##### The right to be involved

Adolescents, parents, and health personnel thought adolescents should have an inherent right to be involved in their treatment [28, 32, 40, 46, 47, 51, 52]. Adolescents wanted to be heard and respected regardless of their age, and to be involved in treatment decisions [32, 40, 47, 50–53]. The right to be involved was essential for adolescents to maintain autonomy [40, 46, 50], for example through their right to refuse health personnel’s proposals [50], including the use of medication [40].

Some health personnel were reluctant to give adolescents control of treatment decisions, in particular due to adolescents diagnoses and lack of ability to express their views [47]. Others pointed out that adolescents with the most severe mental health problems had the greatest need to be actively involved in their treatment [29]. Overall, most healthcare personnel agreed that adolescents have a basic right to express their opinions and to be heard, and they found this was also beneficial to facilitate the treatment [46, 47]. Some clinics had introduced a culture of “no decision without involvement” [29]. This meant, for example, that in these clinics, adolescents were included in all meetings with health personnel. Adolescents expressed a desire to at least retain some control of their treatment and the patient-practitioner relationship [46]. Many adolescents were unaware that they had the right to be involved, or they forgot to pose questions of importance in decision-making processes [42].

##### Collaborative relationship

The majority of health personnel and adolescents emphasized the importance of fostering a collaborative relationship to facilitate the active involvement of adolescents throughout the treatment process [47, 53]. In contrast, an approach characterised by unilateral clinician control was described, where adolescents did not feel included in decisions-making processes, for example about whether they should use medication or by not being invited to meetings concerning their treatment [37, 39, 41, 47, 49, 55]. Health personnel could limit or exert control over treatment choices [37, 41, 46, 47, 49, 51, 55], with some even presenting an ultimatum of “either this treatment, or none” [42, 47]. Consequently, adolescents experienced distress, leading to reduced willingness to engage in their treatment [39, 41, 46, 47, 49, 55]. A collaborative relationship, on the other hand, was characterised by three sub-themes: *information and two-way communication*; *trust*; and *shared decision-making*.

**Information and two-way communication**

Two-way communication was achieved when health personnel provided adolescents with relevant and understandable information, as well as listened to them and were open to take their perspectives into account. Adolescents’ active involvement and motivation in their treatment was facilitated by a collaborative relationship when they received sufficient, understandable, and age-appropriate information [28, 29, 40–42, 46, 47, 50, 55], and where health personnel avoided the use of jargon and technical terms [42]. This included information on treatment options [28, 30], self-care activities [28], and potential benefits of treatment and side-effects of medication [30]. Adolescents should also be provided with information about the right and possibilities to participate in their healthcare, as well as how such involvement may take place [29, 30]. Treatment and areas of decisional conflict goals should be identified [28]. Health personnel thought basic information about treatment and user involvement should be provided prior to hospital admission to establish a dialogue and clarify expectations [29].

Healthcare personnel pointed out that adolescents’ perspectives were not always included in referral letters, although dialogue prior to hospital admission could help to clarify adolescents’ understanding and expectations of treatment goals [29]. To facilitate two-way communication, adolescents needed time to consider and discuss what they believed was the core of their problems, rather than to quickly be diagnosed and expected to follow a standardized clinical pathway [29]. Other adolescents described limited possibilities to voice their opinion, which served as a barrier to user involvement [37, 39, 41, 46, 47, 49, 51, 55]. They did not feel heard [46, 49], but were instead interrupted, ignored, and not asked for their opinions [37, 39, 41, 47, 49, 55]. Adolescents were reluctant to state their opinions when they were only encouraged to express views that were consistent with health personnel’s perspectives and presented in what healthcare personnel considered to be “an acceptable manner” at “an appropriate time” [46, 49].

**Trust**

Relevant and timely information together with communication where health personnel carefully listened to adolescents’ own experiences and shared their professional knowledge, contributed to the second characteristic of the theme *collaborative relationship*, namely *trust* [28, 30, 40, 46].

Adolescents’ active involvement in their treatment was facilitated by adolescent-practitioner relationships where trust had been established [28, 40, 41, 46, 47, 50, 55]. Adolescents, parents and healthcare personnel described a non-judgemental approach, sensitivity to individual preferences [36] and sufficient time was needed in order to establish a relationship where health personnels met adolescents with warmth and thereby showed that they cared about them [30, 50]. Consequently, adolescents felt secure and recognised, and became more actively engaged in their treatment [29, 30, 51]. The opposite was seen when a trust-based therapeutic relationship had not been built. These adolescents did not feel comfortable with expressing their views [42]. Some adolescents suggested that finding the most suitable health personnel for them prior to treatment start, could increase the chance of establishing a good adolescent-practitioner relationship characterised by trust [30]. Establishing a relationship based on trust and acknowledging the needs of adolescents is linked to redistribution of power where adolescents experience that their opinions are taken into account in decision-making processes [33, 40, 53].

Building a connection rooted in trust and acknowledging the needs of adolescents is associated with a shift in power dynamics. This shift ensures that adolescents receive equal attention to their viewpoints, as noted by Boydell in 2010 [33], Hart in 2005 [41], and Wisdom in 2006 [55], which subsequently influences decision-making procedures.

**Shared decision-making**

Shared decision-making was a central part of a collaborative relationship [28, 34, 40, 41, 46–48, 55]. Several studies reported limited extent of adolescent involvement in decision-making processes [37, 39, 41, 46, 49, 51, 55], and some health personnel were reluctant to hand over control to adolescents [47], whereas health personnel in other studies thought adolescents should be involved in decisions affecting treatment and care [28–30]. Treatment decisions encompassed various aspects, including selection of therapist or case manager [28, 47, 50, 53], who adolescents would like to invite to meetings (such as family members) [29, 30, 53], and what information that would (and would not) be shared with their parents [29]. Treatment decisions could also include whether to participate in treatment meetings [29], setting the meeting agenda [29, 30], developing treatment plans [47, 51], and the time, length and frequency of treatment sessions [50, 53]. Different options for adolescents to express their wishes could be provided, such as text message feedback solutions [54]. According to parents, the choice of treatment should be limited by health personnel’s assessment of the severity of adolescents’ mental health conditions and their level of maturity and self-insight [54]. Adolescents felt they were rarely involved in discussions about diagnoses and they perceived this as a way of limiting their involvement in their care [30]. Health personnel’s role as therapists were challenged as they had not learned how to manage shared decision-making [29]. Shared decision-making was facilitated when their knowledge and clinical experience were integrated with adolescents’ knowledge [29].

##### Capacity and support

Capacity and support were found to be essential for adolescents’ user involvement and was described by three main characteristics: *adolescents’ capacity to be involved*; *health personnel’s capacity to facilitate involvement*; and *support for adolescents’ involvement*.

**Adolescents’ capacity to be involved**

Different opinions were found concerning adolescents’ capacity to be actively involved in their healthcare [32, 40, 46, 47, 52]. Some health personnel were concerned about adolescents’ ability to be involved in decision-making processes [46, 47]. They considered some of them to be too young, immature, uninterested [46, 47], or too influenced by mental health conditions such as depression [40, 46, 47]. Some parents said that adolescents were unable to express need for help and lacked insight into their mental health conditions [31]. Both adolescents and parents pointed out that the degree of illness and understanding could limit the capacity to be involved in their healthcare [50], and parents considered pressure to comply with treatment necessary in instances when adolescents were too ill to be involved in decision-making processes [31].

Other health personnel [47], as well as adolescents [32, 52], described adolescents’ interest in and motivation to be involved in decisions affecting their treatment; they wanted to be heard, had clear ideas about their care and had the capacity to make proper judgements. Adolescents had clear opinions about what a treatment plan should contain, who should be involved and what they should be able to decide [30].

**Health personnel’s capacity to facilitate involvement**

Adolescents questioned health personnel’s ability to facilitate involvement because they perceived them as overwhelmed by their workload [52, 55]. Staff shortages forced health personnel to make decisions there and then, instead of giving adolescents time to think about treatment options [42]. Resource constraints did not allow for all adolescents to be involved in shared decision-making to the same extent [50]. Their involvement depended on health personnel’s ability to provide relevant and age-appropriate information, for example on options for medication and treatment, expected outcomes and potential side-effects [28]. Some health personnel consider user involvement a means to reinforce professionalism, as involvement of adolescents in decision-making processes required them to stay updated and confident about their role as professionals [29].

**Support for adolescents’ involvement**

Social and practical support could increase the involvement of adolescents in their mental healthcare [32, 35, 40, 43, 50, 55]. Social support included hearing adolescents, providing them with appropriate treatment choices, providing guidance, and encouraging active involvement in decisions-making processes [28, 40, 47, 48, 50, 55]. User involvement and shared decision-making were easier to implement when adolescents received care and support from their families and social networks, whereas family conflicts served as a barrier to shared decision-making [29, 31]. In order for health personnel to provide treatment options that were also acceptable to adolescents depending on their cultural background, they should explore adolescents’ experiences, views, relationships and support networks [34, 48]. Examples of practical support included shared decision-making worksheets to facilitate adolescent involvement [28] and means to enable adolescents to come to consultations as limited transport options could reduce their ability to be actively involved [52].

##### Outcomes of user involvement

Adolescents reported that active involvement in their treatment was associated with higher treatment attendance rates [50] and treatment continuation, as opposed to drop-out [41, 50]. They felt empowered when they were involved in shared decision-making processes [42]. Contrary to this, adolescents who were not actively involved, described passive compliance and disengagement from their treatment [32]. Some parents described adolescents as more independent and taking better care of themselves when they were actively involved in their treatment, whereas the opposite resulted in poorer treatment outcomes [31]. Adolescents who were pushed to do something tended to disagree with any suggestion, they pretended to agree, became silent or responded “I don’t know” instead of engaging in treatment sessions [31]. Those who were not actively involved, became passive recipients of treatment [30]. Several studies described distress and reduced willingness to be involved in treatment amongst adolescents who were pressured or who felt ignored [39–41, 47, 49, 55]. Health personnel experienced that involvement taking place at an early stage supported adolescents in becoming more motivated and it limited the need for involuntary treatment [29].

#### User involvement at the organizational level

The update search did not identify new articles reporting on user involvement at the organizational level and the themes from thematic synthesis are therefore equivalent with the initial review [15]. We provide a summary of the main content from the two themes, *involvement outcomes relevant to adolescents’ needs*; and *conditions for optimal involvement*.

##### Involvement outcomes relevant to adolescents’ needs

Involving adolescents at an organizational level contributed to use of terminology and design of services relevant to adolescents [35, 48, 54]. Their involvement in designing and implementing interventions and therapy reflected their experiences and needs [38], and improved treatment outcomes through increased relevance, appropriateness, and acceptability [31, 34, 35, 52]. Moreover, adolescents’ perspectives could contribute to improve health personnel’s training [43], create treatment environments better adapted to meet adolescents’ needs [33], and support their peers in identifying personal goals [37]. The involvement of adolescents at an organizational level also fostered a sense of empowerment, which positively influenced their recovery [34, 35].

##### Conditions for optimal involvement

Involvement of adolescents at the organizational level required health personnel to be open to adolescents’ perspectives [48], to ensure clarity of roles [35, 37, 52], and leaders to formally acknowledge and encourage such involvement [35, 37]. It was crucial to provide adolescents with information about available services and potential projects they could participate in [37, 51], while they also had the autonomy to choose their preferred level of involvement [37, 48, 51, 52]. Adequate skills training should be provided to support adolescents to participate [35, 37]. Adolescents’ personal experiences with mental health services contributed to optimize their involvement, particularly in roles such as peer consultants who directly interacted with other adolescents [35, 52]. Moreover, involving adolescents from diverse social, ethnic, gender, and sexual orientation backgrounds enhanced diversity and broadened the scope of relevant services for a wider range of adolescents [33, 35].

### Characteristics of quantitative studies

Quantitative methods were used in eight studies. Among these, seven reported on user involvement at the individual level [56–62], while one focused on user involvement at the organizational level [63]. Various research designs were used, including a single randomized controlled trial [60]; a non-randomized comparative study [59]; two longitudinal prospective cohort studies [56, 57]; a cohort study using pre- to post-assessment [60]; and three cross-sectional surveys [58, 62, 63]. One of the cross-sectional surveys employed repeated measures for some participants [63]. There was a considerable degree of heterogeneity among the studies. Additional characteristics of the studies can be found in Table 6.

**Table 6:** Effectiveness of user involvement in adolescent mental healthcare

Reference	Study design	Participant characteristics	Intervention, study setting	Trial/study arms^a^	Results	Internal and external validity assessment^b^
Jager 2014 [56], Netherlands	Longitudinal prospective cohort study	Age: 12–18 years, female 65%, adolescents who signed up for psychosocial care (76% in mental health care)	**Psychosocial care**, mostly delivered by a mental healthcare organization (76%). Care with patient-centered communication, including shared decision-making Duration 3 months Specialist healthcare services	T1 (baseline): *N = *416 T2 (3mo.): *n = *211 (51%) (min. 2 appointments)	Shared decision-making on the **Consumer Quality Index** (CQI) at 3 months: Adolescents who considered shared decision-making to be important (expectations), but experienced it to less extent, had lower degree of improved understanding of mental health problems and how to handle them, compared to those who had agreement between expectations and experiences (OR 4.2, 95% CI 1.7–10.8, p < 0.01)	**Internal validity**: Overall risk of bias: high Selection, performance, detection and attrition bias: high. Reporting and other forms of bias: low **External validity**: Equally pragmatic and explanatory
Jager 2017 [57], ^**c**^ Netherlands	Longitudinal prospective cohort study	Age: 12–18 years (x̄ = 15.2, SD1.7) female 61%, adolescents who signed up for psychosocial care (77% in mental health care)	**Psychosocial care**, mostly delivered by a mental healthcare organization (77%). Care with patient-centered communication, including shared decision-making. Intervention duration: 6 months Specialist healthcare services	T1 (baseline): *N = *416 T2 (3mo.) + T3 (1year): *n = *315(76%)	**Strengths and Difficulties Questionnaire** (SDQ) with changes in Total Difficulties Score (TDS) from T1 to T3: Experience of shared decision-making associated with larger improvement in TDS scores, irrespective of adolescents’ expectations. Unmet shared-decision making communication needs associated with lower improvement in self-confidence (p < 0.001)	**Internal validity**: Overall risk of bias: high Selection, performance, detection and attrition bias: high. Reporting and other forms of bias: low **External validity**: Equally pragmatic and explanatory
Nolkemper 2019 [58], Germany	Cross-sectional survey	Age: 12–18 years (x̄ = 14.8, SD1.5), female 42%, adolescents who have been hospitalized for mental health conditions	**Psychiatric treatment** Child and adolescent psychiatry medical college & child and adolescent psychiatry university hospital	Experience of participation in psychiatric treatment: *N = *114	Self-developed questionnaire focusing on feeling of being able to participate in decision-making (6 items, Likert scale): Yes, very much: 12% Yes: 40% Partially: 25% Not really: 13% Not at all: 10% No significant age, gender or clinic differences	**Internal validity**: Overall risk of bias: high Selection, performance, detection, attrition and other forms of bias: high. Reporting bias: low **External validity**: Equally pragmatic and explanatory
Simmons 2017 [59], Australia	Non-randomized comparative study	Age: 16–25 years (x̄ = 17.8, SD2.9), female 63%, adolescents who attended a youth mental health service clinic	**Peer workers** engaged with adolescents during intake assessment and online shared decision-making tool, prior to individual counseling session with a clinician Historical comparison group without peer workers and online shared decision-making tool E-health in primary & secondary care	I: *n = *149 Response to SDMQ-9: *n = *78 (52%) C: *n = *80 Response to SDMQ-9: *n = *61(76%)	**Shared Decision Making Questionnaire** (SDMQ-9) (clinician rated) on day 1: In favor of the intervention group (p = 0.015), but limited clinical effect (mean difference 2.4 on a 54 point scale)	**Internal validity**: Overall risk of bias: high Selection, performance, detection and attrition bias: high. Reporting and other forms of bias: unclear **External validity**: More pragmatic than explanatory
Simmons 2017 [60], Australia	Cohort study with pre- to post-assessment	Age: 12–25 years (x̄ = 18.5, SD3.4), female 82%, depression (PHQ-9): mild (min.5 points)(18%), mild–moderate (26%), moderate–severe (56%)	**Online decision aid** to help adolescents make decisions in line with evidence and their personal preferences and values Primary care	T1(before decision aid): *N = *66 T2(after decision aid): *n = *57 (86%) T3(8 weeks): *n = *48 (73%)	**Patient Health Questionnaire** (PHQ-9) from T1 to T3: mean reduction of 2.7 points (95% CI, 1.3;4.0) **Decisional Conflict Scale** (DCS) from T1 to T2: mean reduction 17.8 points (95% CI 13.3;22.9, p < 0.001)	**Internal validity**: Overall risk of bias: high Selection, performance, detection, attrition and reporting bias: high. Other forms of bias: low **External validity**: More pragmatic than explanatory
Walker 2010 [63], USA	Cross-sectional and repeated measures survey	Age 14–21 years (x̄ = 16.2,SD1.7), female 43%, mental health difficulties: ADHD, depression, bipolar disorder, PTSD, ODD, conduct disorder	**Testing of a Youth Empowerment Scale–Mental Health** (YES–MH), adapted from the Family Empowerment Scale (FES), services provided by multiple child- and family-serving agencies, primary & secondary care	T1 (baseline): *N = *185 T2 (6 weeks): *n = *60	Results based on exploratory factor analysis of YES–MH suggest three levels of empowerment: a) system: confidence & capacity to help providers improve services and help other youth with emotional/mental health difficulties b) services: confidence & capacity to work with service providers to select and optimise services c) self: confidence & capacity to cope with or manage one’s own condition Positive correlation between YES-MH and a 6-item Participation in Planning Scale (PPS)(*p* < 0.01)	**Internal reliability**: very good for both YES-MH (Cronbach’s alpha 0.85 – 0.91) and PPS (0.90) **Test–retest reliability** good for all three sub-scales of YES-MH (p < 0.01). No other forms of psychometric tests were applied
Walker 2017 [61], USA	Randomized controlled trial	Age: 12–18 years (x̄ = 14.2, SD1.3), female 42%, serious mental health problems	**Wraparound**: team working with adolescents, their family members and the family’s social support network, determining the primary needs, service and support strategies to be included in the care plan **AMP**: Achieve My Plan, enhances Wraparound through multi-system involvement with caregivers and service providers Outpatient CAMHS	I: Wraparound with AMP: *n = *35 C: Wraparound without AMP: *n = *20	Primary outcomes: **Youth Participation in Planning Scale** (YPP): Youth participation in preparation and planning in favor of the intervention at 3–4 weeks and 10–12 weeks (*p* < 0.01). Accountability in favor of the intervention at 3–4 weeks (*p* < 0.03), but not at 10–12 weeks (*p* = 0.10) **Youth Empowerment Scale** (YES): No significant effects Secondary outcome: Intervention group participants were 2.35 times more likely to rate care planning meetings as much better than control group participants (*p* < 0.001)	**Internal validity**: Overall risk of bias: high Performance bias: high Selection, detection, reporting and other forms of bias: unclear **External validity**: More pragmatic than explanatory
Zerbe 2021 [62], Germany	Cross-sectional survey	Age: 9–18 years (x̄ = 14.1), female 63%, mental health difficulties: anxiety and compulsive disorder (30%), depression (23%), eating disorders (15%), ADHD (10%), psychotic episode (4%)	**Psychiatric treatment** at two university hospitals and three specialist clinics	Experience of participation in psychiatric treatment: *N = *81	**Primary outcome**: Experience of overall treatment participation (4.17, SD0.46) was lower than desire for participation (3.41, SD 0.74) measured on a 5-point Likert scale. **Secondary outcomes**: Received information (3.80, SD0.89) was lower than desire for infromation (4.40, SD0.55), involvement in decision-making was lower (2.42, SD1.02), compared to desire for involvement (3.28, SD0.74), and involvement in treatment decisions was lower (3.41, SD1.11), compared to desire for such involvment (4.11, SD0.79). All p < 0.001	**Internal validity**: Overall risk of bias: high Selection, performance, detection, attrition: high. Reporting bias and other forms of bias: unclear **External validity**: More pragmatic than explanatory

^a^I = Intervention, C = Control

^b^Internal validity: Cochrane Collaboration’s guidelines for risk of bias assessment [22]. External validity: The PRECIS tool for assessing studies on a pragmatic-explanatory continuum was used [23]. Validity assessment for Walker 2010 [63] focuses solely on criteria of relevance to psychometric tests

^c^Jager 2017 [57] builds on the same data as Jager 2014 [56], but assesses different outcomes and includes long-term follow-up

### Quality assessment of quantitative studies

All seven studies focusing on user involvement at the individual level were deemed to have a high risk of bias, based on guidelines provided by the Cochrane Collaboration [20]. Four studies were classified as more pragmatic than explanatory [59–62], according to the PRECIS tool [23]. The remaining three were equally pragmatic and explanatory [56–58]. Further details are presented in Table 6.

### Effectiveness of user involvement

Effectiveness of user involvement report on the quantitative studies included in the review. Only one additional study [62] was identified in this updated systematic review, reporting on the effectiveness of user involvement at the individual level.

#### User involvement at the individual level

A few studies included in the systematic review published in 2022 [15] assessed the effectiveness of additional support to facilitate involvement of adolescents in their care [59–61]. The results of a randomized controlled trial suggested that a team assisting adolescents with severe mental health issues helped to support their involvement in treatment planning in the short (3–4 weeks) and longer (10–12 weeks) term [61]. Adolescents who received support were more than twice as likely to view care planning meetings positively compared to a control group. A non-randomized controlled trial found the use of peer workers together with an online shared decision-making tool before counselling sessions resulted in a small effect in adolescents’ perceived decision-making measured using the Shared Decision-Making Questionnaire (SDMQ-9) [59]. The results of a cohort study suggested that an online tool designed to assist adolescents with depression to make decisions in line with their values and research-based evidence was associated with a significant reduction in depression scores (PHQ-9) by 8 weeks, although the clinical significance of the change was uncertain (mean change 2.7 points, 95% CI 1.3–4.0) [60]. Improvements measured using the Decisional Conflict Scale (DCS) after using the tool were significant (mean change 17.8 points, 95% CI 13.3–22.9). Two longitudinal cohort studies found shared decision-making to be helpful in the short term to support adolescents’ ability to handle their mental health better [56], and to manage their difficulties and strengthen their self-confidence in the longer term [57].

The updated review adds knowledge based on data collected in a single cross-sectional study aiming to assess user involvement at the individual level, with 81 adolescents recruited from five German child- and adolescent psychiatric clinics [62]. The study assessed three dimensions of adolescents’ involvement in their mental healthcare in line with Charles, Gafni and Whelan’s model [64]: information exchange, reflection and discussion, and decision-making. Adolescents reported being significantly less involved in their treatment than they desired, for all three dimensions. Feeling involved in their treatment was strongly correlated with patient satisfaction. Those who had long-lasting illness were more interested in taking on an active role in their treatment, whereas those with more severe illness were less interested in doing so. Age did not determine adolescents’ willingness to be actively involved in their treatment.

#### User involvement at the organizational level

No additional studies were found in the update review to shed light on user involvement at the organizational level. This leaves only a single study suggesting empowerment of adolescents to support their confidence and capacity to work with service providers, to help to improve the services and to support other adolescents with mental health difficulties [63]. The authors described empowerment as “a common idea of subordinated people gaining or attaining the capacity to control their own lives and to influence the community and social structures that affect their lives” [p.52].

### Safety associated with user involvement

No additional studies were found in the updated review to add to the evidence focusing on the safety of user involvement collected from two studies identified in the systematic review published in 2022 [15]. Findings from the systematic review published in 2022 suggested that some health personnel considered involvement in decision-making to be a potential threat to patient safety [45], whereas other health personnel were concerned about breach of confidentiality and barriers to recovery among adolescents who served as adolescent consultants who supported other adolescents with mental health challenges [35].

## Discussion

While the existing body of literature remains somewhat dispersed, the results suggest that adolescents, parents, and healthcare personnel consider user involvement to be beneficial to facilitate mental health treatment tailored to meet the needs of adolescents. In their understanding, user involvement enhances the relevance, appropriateness, and acceptability of the treatment, thereby contributing to increased treatment attendance, higher treatment satisfaction, and improved treatment outcomes. According to the results, both individual and organizational-level user involvement promote empowerment and recovery, although the literature primarily emphasizes user involvement at the individual level [15]. We found that limited evidence exists regarding the effectiveness of user involvement, although results of quantitative studies indicate a correlation between user involvement and patient satisfaction. Additionally, the results show that adolescents tend to exhibit a more positive perception of their treatment plans, strengthened self-confidence, and enhanced resilience in facing life challenges when user involvement is integrated into mental health treatment. No studies focus explicitly on safety concerns associated with user involvement.

In line with the increasing recognition of user involvement, shared decision-making has gained broad support across healthcare services over the past decade and is recommended in clinical guidelines [10, 65]. A summary of shared decision-making over the last 21 years points to an international paradigm shift towards person-centered services [66]. During this period, several theoretical models and tools have been developed to describe the elements of a shared decision-making process. Key features for implementing shared decision-making have been identified, such as leadership, coordination, training, enabling users to participate in decisions, and redesigning care pathways. However, there is still a gap between existing research-based knowledge and routine practice in clinical settings. To enhance user involvement in decision-making processes, a more extensive understanding is needed concerning how shared decision-making functions across different groups and settings [66].

There is still no internationally agreed view on what shared decision-making entails [67]. The results of our updated systematic review could thus complement the current understanding of mental health services for adolescents. We found that shared decision-making, together with trust, information exchange, and two-way communication, contribute to a collaborative relationship. Therefore, shared decision-making should not be viewed in isolation or stand alone as a methodology. It should rather be understood as part of a comprehensive treatment approach that emphasizes the importance of the relationship between adolescents and health personnel. Our findings are supported by a literature review focusing on shared decision-making within the context of severe mental illness in adults [68]. The current review identified a reciprocal relationship between shared decision-making and the therapeutic alliance, highlighting the need to emphasize user preferences and relationship-building in clinical practice. Recognition of adolescents’ preferences and adapting treatment approaches accordingly, entails a shift from a power-dependent relationship to a more balanced partnership [30, 36]. In addition to the unique development needs of adolescents, it is important to recognize that user involvement in healthcare has relevance across the lifespan. Findings from adult research highlight that core elements of healthcare, such as shared decision-making, trust, and two-way communication, are essential for person-centered care [69] underscoring the universal nature of these components. Whether in adolescence or adulthood, healthcare systems should be designed to promote user involvement, as it might enhance not only treatment satisfaction and adherence, but also empowerment and recovery. By acknowledging these similarities, we can adopt a lifespan perspective that ensures that user involvement remains a cornerstone of healthcare across all stages of life.

The significance of the therapeutic alliance has been well documented in prior research, not only in studies related to forms of user involvement. Research aimed at investigating effective factors in therapy has described an emotional bond founded on trust and understanding of the user as a prerequisite for therapeutic effectiveness [70]. A partnership between health personnel and adolescents can, in addition to creating the context for user involvement and treatment efficacy, be understood as essential to support adolescents’ inherent right to express their views and have them duly considered as limited opportunities. This partnership is linked to international rights and legislation as the cultivation of user involvement creates the frame for adolescents to be adequately consulted and express their views [71]. The results of this updated systematic review reveal notable variations in how user involvement is practiced and indicate that these rights are still not adequately fulfilled. Furthermore, it is evident that adolescents have clear opinions about their own treatment and their capacity to participate in decision-making.

The extent and severity of mental health problems are often used as justification for limiting involvement, underscoring the importance of considering adolescents individualities concerning their wishes to participate and recognizing that adolescents with enduring or severe illness seem to benefit more from active involvement in their treatment [29, 62]. Resource limitations have been identified as a barrier for health personnel to implement user involvement, highlighting the need to incorporate practical support for user involvement and prioritize training in health personnel’s skills to promote collaborative relationships and support adolescents’ involvement. Moreover, online tools and shared decision-making tools have been found to be useful to assist decision-making processes [59, 60]. They are potentially cost-effective strategies to promote adolescents’ involvement in their care.

The systematic review published in 2022 found a lack of literature exploring safety issues of how adolescents may be involved to improve patient safety. Our updated search did not identify new research evidence evaluating safety concerns associated with user involvement in mental healthcare for adolescents. A research gap exists to identify safety issues associated with user involvement at the individual and organizational level. Adolescents are at a stage where their autonomy is growing, but their ability to fully participate in decision-making may still need further development [31], making it essential to balance their involvement with considerations of safety and support. Adressing this dynamic and ensuring that adolescents’ right to involvement is recogniced in helathcare settings remains a priority. Further research is needed to determine how to safely implement user involvement for adolescents with variable capacities. Nevertheless, patient experiences are positive associated with patient safety and clinical effectiveness [72]. The qualitative literature synthesized in this updated systematic review informs future research on patient safety for adolescents in mental healthcare. Our findings suggest that patient safety for adolescents in mental healthcare may be related to patients’ experiences of having formal rights to be involved. It includes a collaborative relationship characterized by sharing information and two-way communication, trust, shared decision-making, and ensuring that health personnel have the expertise and capacity to involve patients. Appropriate and robust quantitative studies are needed to determine whether these dimensions of user involvement are associated with patient safety outcomes.

### Strengths and limitations

The use of multiple databases, a wide range of search terms and a youth co-researcher involved in the analytic process are considered as strengths. Still, there is a possible oversight of relevant studies due to the lack of standardized search terminology in the field and the heterogeneity of identified studies due to wide inclusion criteria. Furthermore, in the time lag between literature searches, the analytic processes, writing the article and the journal’s review processes means that the most recent publications may not be included. An additional review in co-production in child and adolescent mental helth services has been noted, which identified only two studies of poor research quality [73]. This review highlights the limited literature in this area and the need for futher research. Our systematic review complements it by providing a broader synthesis of user invovlement, adressing both individual and organizational levels. It thus expands knowledge and contributes with insight and an overview of adolescents’ involvement in the field of mental healthcare. Limitations in the identified existing literature prevent us from providing clear recommendations related to different groups of adolescents or issues related to safety. Similarly, there is limited published literature on user involvement at the organizational level, resulting in a limited update in this area compared to the previous review [15]. While this study defines adolescence as the age range from 13 to 18 years for the purposes of analysis, we acknowledge that this boundary is subject to debate, with developmental science and global perspectives often extending adolescence to beyond the age of 18.

## Conclusion

This updated systematic review offers an updated insight into user involvement in adolescents’ mental healthcare, both at the individual and organizational level. Adolescents, parents, and health personnel emphasized adolescents’ inherent right to be involved in their treatment and embraced shared decision-making as a means to facilitate user involvement and person-centered care. There were insufficient studies using quantitative research designs to determine the effectiveness of user involvement. However, the evidence gathered from qualitative studies suggests actively involving adolescents in their treatment contributed to greater motivation for treatment, higher attendance rates and treatment continuation. User involvement contributed to reduced need for involuntary treatment and reduced drop-out rates. A collaborative relationship served as a facilitator to user involvement, characterized by provision of information exchange, two-way communication, establishing a trusting relationship, and applying shared decision-making. Moreover, user involvement depended on adolescents’ desire and capacity to be involved, health personnel’s capacity to facilitate involvement, and sufficient social and practical support to enable adolescents’ involvement.

Although user involvement in adolescents’ mental healthcare has become increasingly common, challenges persist in translating research-based knowledge into routine clinical practice. Moreover, the field of user involvement still lacks clear definitions and standardized terminology. We suggest user involvement should be integrated into any mental health treatment provided for adolescents. Furthermore, user involvement should emphasize adolescents’ preferences and a collaborative relationship which incorporates shared decision-making. User involvement has the potential to enhance the quality of care provided for adolescents with mental health challenges. However, translating these principles into effective practice requires ongoing commitment, addressing resource limitations, and focusing on involvement both at the individual and organizational level.

### Implications and further research

- Healthcare systems and institutions should align with international rights and national legislation to ensure that adolescents’ rights to express their views and have them considered are upheld. This involves actively seeking input from adolescents in decisions affecting their mental healthcare.
- Healthcare organizations should develop and provide practical support to implement user involvement. This should include training for health personnel to enhance their skills in promoting collaboration and supporting adolescents’ involvement.
- Guidelines for the implementation of user involvement at the individual and organizational level should be established. This would contribute to translating research-based knowledge into routine clinical practice.
- Online tools and decision-making tools with age-appropriate information are recommended to support user involvement and shared decision-making.
- Future research should investigate safety issues associated with user involvement both at the individual and organizational level. More robust quantitative studies are also needed to assess the effectiveness of user involvement in adolescents’ mental healthcare.

## Data Availability

The research materials can be accessed by contacting the corresponding authors.

## Abbreviations

ADHD: Attention Deficit/Hyperactivity Disorder
CASP: Critical Appraisal Skills Programme
DCS: Decisional Conflict Scale
PRECIS: The Pragmatic Explanatory Continuum Indicator Summary
PRISMA: Preferred Reporting Items for Systematic Reviews and Meta-Analyses
SDMQ-9: Shared Decision-Making Questionnaire
CI: Confidence Interval
STROBE: STrengthening the Reporting of OBservational studies in Epidemiology
UN: United Nations
